# A Cluster Based Localization Scheme with Partition Handling for Mobile Underwater Acoustic Sensor Networks

**DOI:** 10.3390/s19051039

**Published:** 2019-02-28

**Authors:** Tariq Islam, Yong Kyu Lee

**Affiliations:** Department of Computer Science and Engineering, Dongguk University-Seoul, Seoul 04620, Korea; tariqislam20@yahoo.com

**Keywords:** underwater acoustic sensor networks, localization, mobility, GPS, clustering, data-tagging

## Abstract

Many applications of underwater sensor networks (UWSNs), such as target tracking, reconnaissance and surveillance, and marine life monitoring require information about the geographic locations of the sensed data. This makes the localization of sensor nodes a crucial part of such underwater sensing missions. In the case of mobile UWSNs, the problem becomes challenging, not only due to a need for the periodic tracking of nodes, but also due to network partitioning as a result of the pseudo-random mobility of nodes. In this work, we propose an energy efficient solution for localizing nodes in partitioned networks. Energy consumption is minimized by clustering unlocalized partitioned nodes and allowing only clusterheads to carry out a major part of the localization procedure on behalf of the whole cluster. Moreover, we introduce a retransmission control scheme that reduces energy consumption by controlling unnecessary transmission. The major design goal of our work is to maximize localization coverage while keeping communication overheads at a minimum, thus achieving better energy efficiency. The major contributions of this paper include a clustering technique for localizing partitioned nodes and a retransmission control strategy that reduces unnecessary transmissions.

## 1. Introduction

Underwater acoustic sensor networks (UWASNs) offer a vast number of applications including, but not limited to, mineral exploration, underwater installation monitoring, pollution monitoring, disaster prevention, surveillance, reconnaissance, and target tracking [1]. The majority of these and other UWASN applications require location information about sensor nodes in order to relate the sensed data to their geographical location. For instance, in defense related applications the absolute location of sensor nodes can support the detection and tracking of an intruding object [2]. In marine life monitoring, absolute location information of sensor nodes can help to detect and track animals and observe their behaviors. In pollution monitoring, such as oil spills and ocean trash, location information helps determine the extent of the affected area and concentration of pollutants in different parts of the affected area [3]. Similarly, in cases of natural disaster such as a tsunami, the location of underwater tectonic plate movement can be determined. The location along with sensed intensity enables the estimation of the impact on coastal areas. As in all these applications, data collected by sensor nodes must relate to the geographical location from where it is collected, thus the localization of sensor nodes becomes an area of pivotal importance.

However, unlike terrestrial networks where GPS is used to collect location information, underwater sensor networks are constrained by the unavailability of GPS. The radio waves used by GPS cannot penetrate more than a few meters into water [4], thus rendering it useless for underwater applications. Additionally, due to the free mobility of sensor nodes with water currents, UWSNs are prone to network partitioning, which may result in a smaller localization ratio. Improvement in the localization ratio requires additional communication, thus reducing the energy efficiency of the network. The above given constraints dictate the need for underwater localization mechanisms that should be able to (a) localize the maximum number of nodes, (b) efficiently handle mobility-induced issues (e.g., network partitioning) and (c) keep energy consumption and communication overheads to a minimum.

In this work, we propose an iterative clustering based localization protocol for mobile underwater sensor networks. We assume that our network consists of two types of nodes: (a) Two or more GPS-enabled nodes and, (b) Ordinary sensor nodes. The first with two or more GPS-enabled nodes, as well as ordinary sensor nodes. We propose that there are backup GPS nodes which perform as ordinary sensor nodes in normal circumstances. However, if the main GPS node goes down, one of the GPS enabled nodes takes charge. This can be done as follows. At the start of every localization cycle, the main GPS node communicates with other GPS nodes to announce that it is alive. The "alive message" from the GPS node contains a sequence in which the backup nodes will take charge if the main GPS node goes down. If backup nodes do not hear from the main GPS node for a certain duration of time, the top node in the sequence takes control. 

GPS nodes know their initial absolute coordinates at the time of deployment. Moreover, they are equipped with the necessary apparatus for tracking their movement in 3D space (e.g., gyroscope). Therefore, these nodes can update their coordinates underwater with the help of displacement information acquired through the movement tracking apparatus and their initially known position. In order to refresh the GPS coordinates, these nodes periodically rise to the water surface, and get fresh GPS coordinates. Unlike the GPS node, ordinary sensor nodes are not equipped with GPS and movement tracking devices. Therefore, these nodes rely on the GPS node (or any localized ordinary node) to estimate their absolute geographical coordinates. The GPS node initiates the localization process by flooding a beacon into the network. The beacon contains the current GPS coordinates of the sender. An ordinary sensor node X can estimate its absolute coordinates with the help of an already localized node Y (e.g., GPS node or a localized ordinary node) if it knows the following two pieces of information:

1. The relative position of node X with respect to node Y.
2. The absolute position of node Y.

Every node Y, when localized, announces its absolute coordinates by broadcasting a beacon. The beacon contains node Y’s absolute coordinates. On reception of the beacon, node X can use Time Difference of Arrival (TDoA) [5] to estimate its position relative to node Y. 

Our main contributions are as follows:

1. A clustering based mechanism to localize partitioned nodes. Only clusterheads send localization requests on behalf of their cluster. This results in reduction in contention, communication overheads, and energy consumption. Further, this yields a high localization ratio.
2. A retransmission control mechanism used by nodes to determine whether, after being localized, they need to retransmit a beacon. This mechanism reduces communication overheads by restricting unnecessary transmission.
3. In contrast to other mechanisms, we allow only the GPS node to respond to localization requests. This results in a sharp decrease in responses to localization requests and gives better localization accuracy by avoiding error accumulation.

The rest of the paper is organized as follows. Section 2 details the related work. Section 3 explains the methodology in detail. Section 4 details the simulation setup, while Section 5 presents the results and provides a discussion. Section 6 concludes the paper.

## 2. Related Work 

Reverse localization scheme [6] is an event driven localization scheme in which nodes are localized to report a sensed phenomenon. Upon detection of an event, underwater nodes transmit messages towards the surface anchor nodes. The anchor nodes use Time of Arrival (ToA) to estimate distance of the sender. The anchor nodes send the distance estimates along with the anchor node’s absolute location to the sink node, which can estimate the absolute position of the sensor node based on its distance from three different anchor nodes and the absolute positions of the anchor nodes. This scheme may incur high communication overheads and thus high energy consumption as the localization of every single sensor node will require at least four transmissions: one from the sensor node to the anchor node and one transmission from each anchor node to the sink node. Moreover, the depth of sensor nodes also plays a role in energy consumption as deeper nodes need more transmission power to transmit to the surface anchor nodes.

The area-based localization scheme (ALS) proposed in [7] is a location estimation scheme which estimates the location of a node within a certain area rather than its exact location. In ALS, reference nodes transmit beacons periodically by varying transmission power according to the requirements. Based on the ranges used by reference nodes, the network is partitioned into non-overlapping sub-areas. Each unlocalized node maintains a list of anchors from which it has received beacons and the corresponding transmission power used to transmit beacons. This information, along with sensed data is forwarded to the sink node which can estimate the certain area within which the node resides. The major drawback of ALS is its non-suitability for applications which require the exact localization of a phenomenon (e.g., target tracking, intrusion detection etc.). Moreover, the transmission of beacons by multiple anchor nodes increases the communication overhead and thus energy consumption. 

The silent positioning scheme [8] proposed in the UPS model is an example of localization schemes that are designed for stationary underwater sensor networks. This scheme works for one-hop underwater networks. Location estimation is carried out with the help of four anchor nodes, which transmit beacons sequentially. The underwater ordinary sensor nodes do not send any localization messages, thus UPS is silent. The communication cost of UPS is low thus making it more energy efficient. The silent positioning scheme is a range-based scheme and uses TDoA for range estimation, therefore it does not require time synchronization. A drawback of UPS is that it can localize only those nodes which are located inside the area enclosed by the anchor nodes. Moreover, the positions of anchor nodes should be fixed and known to sensor nodes in advance.

Underwater Sensor network Positioning (USP) [9] is a projection based scheme for the localization of underwater sensor nodes in sparse 3D underwater networks. USP achieves the task of localization of sensor nodes in two phases: a pre-distribution (or pre-deployment) phase during which each sensor node is preloaded with a unique ID and its position at the time of deployment. Moreover, three nodes are randomly selected as anchor nodes. The distribution phase repeats iteratively. Each iteration is divided into three main periods ∆B, ∆C and ∆S during which a sensor node makes a local broadcast of position information, computes it position information and sleep respectively. As in each iteration, each sensor node broadcasts any new position information that it has, a high communication overhead is incurred that increases energy consumption. Moreover USP is designed for static networks and does not address mobility related issues like network partitioning. 

In [10] a multi-stage localization scheme called 3 dimensional localization algorithm for underwater sensor network localization or 3DUL is proposed for three dimensional underwater networks. 3DUL is divided into two different phases: a Ranging phase which involves a two way message exchange to estimate the propagation delay between a sensor node and anchor nodes. This enables the sensor node to estimate its distance from its neighboring anchors. Once distances to at least three anchors are estimated, the second phase (e.g., "projection and dynamic trilateration phase") starts. During this phase the sensor node locates itself using the information from phase one and becomes an anchor node. 3DUL does not address mobility induced issues such as network partitioning. Moreover it involves a high degree of message exchange. 

Localization with Directional Beacons (LDB) [11] is an anchor-free positioning scheme. LDB employs an autonomous underwater vehicle (AUV) equipped with a directional acoustic transceiver as a mobile beacon. The AUV broadcasts beacons. Sensor nodes passively listen to the beacons and calculate their coordinates based on the coordinates of the autonomous vehicle at the time of entry and exit from the range of the sensor node. Localization accuracy of LDB is not very high, and is affected by both vertical and horizontal errors depending on the frequency of AUV messages [12].

In [13] a multi stage localization scheme called AUV-aided underwater localization scheme is proposed. In this multi stage scheme some nodes are localized by the AUV in the first stage. The nodes localized in the first stage become reference nodes for localizing the remaining nodes in the network in subsequent stage(s). All nodes that are localized in a particular stage, broadcast beacon thus increasing communication overheads and localization errors as the number of hops between the AUV and the node being localized increases. Moreover communication overheads and energy consumption may also be high as the scheme does not define an optimal path for AUV traversal in the network. Furthermore, the scheme does not define any mechanism to handle network partitioning.

AUV-Aided Localization (AAL) [14] is a localization scheme for hybrid 3D underwater sensor networks. An AUV used as a mobile beacon broadcasts a wakeup message to declare its presence. Upon reception of the wakeup message, the receiving sensor node estimates its distance from the AUV and sends a localization request. The AUV responds to the localization request by sending a response message which contains the absolute coordinates of the AUV. The number of necessary response messages differs according to the localization technique being employed (e.g., triangulation or bounding-box). The localization error of AAL may be improved by frequent location calibration, however this may deplete the AUV’s battery quickly. Moreover, the three way message exchange between sensor nodes and the AUV incurs high energy consumption. 

The reactive localization algorithm (RLA) [15] localizes nodes upon detection of an event. The localization of a node is carried out in two steps. In the first step, sensor nodes broadcast a hello message (containing the ID and energy level of the node) for its neighbors. At least four anchor nodes are selected using the K-node coverage algorithm. The second step involves the reactive localization of the sensor node. In this step, sensor nodes send localization requests upon the detection of an event. Upon reception of a localization request, anchor nodes reply with their location information. The sensor node hence localizes itself by quadrilateration. RLA incurs high communication overheads and has low energy efficiency due to the additional process for the anchor nodes’ localization. Moreover, error accumulation also exists.

A Two Phase Time Synchronization Free Localization Algorithm (TP-TSFLA) [16] is a distributed two stage localization. During stage one, all nodes that can receive beacon from the reference node are localized. During stage two, the localized nodes act as reference nodes to localize the remaining unlocalized node. The unlocalized nodes increase their range to reach any localized node that responds with beacon. This scheme can incur high communication overheads, and thus high energy consumption, due to the fact that a particular unlocalized node may receive unnecessary beacons from a large number of reference nodes. In this text, this technique is referred to as simple two-stage localization without clustering, or 2S. 

**Figure 1:**
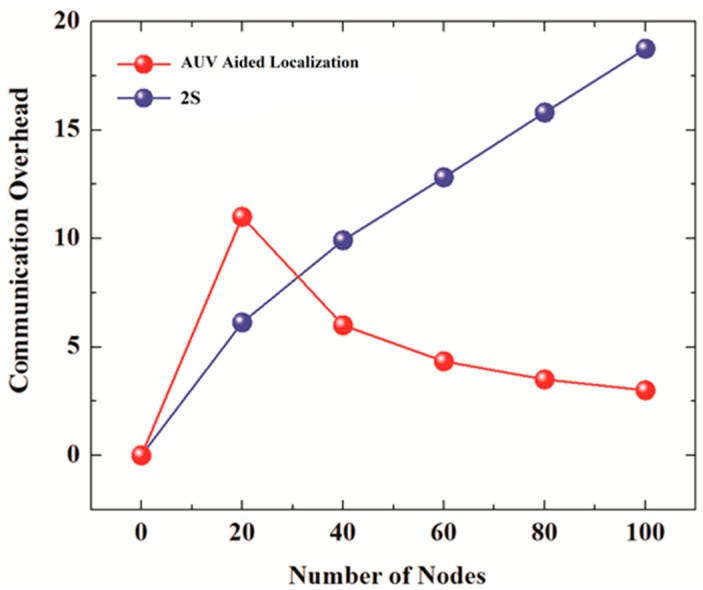
Communication overhead of 2S and AUV Aided Localization clustering.

2S does not define any clustering mechanism to localize partitioned nodes. It assumes that all unlocalized nodes transmit localization requests proactively if they remain unlocalized until a certain threshold time. All those nodes that receive a localization beacon in response to their request are localized, whereas all unlocalized nodes that could not receive a beacon send a localization request again with increased transmission power. Every node keeps increasing its transmission power until it is localized or if power cannot be increased due to hardware limitation. Moreover, 2S also assumes that any localized node that receives these localization requests responds by transmitting a beacon. Because every unlocalized node transmits localization requests and every unlocalized node responds to the request, a huge communication overhead is created. Figure 1 shows the communication overhead using this technique for 20,40,60,80, and 100 nodes. The high communication overhead incurred by this technique not only wastes scarce underwater bandwidth, but also increases energy consumption. Moreover, as the number of nodes increases from 20 to 100, the communication overhead increases from around 6 to 18. This sharp increase points to the lack of scalability of this scheme. 

## 3. Methodology

### 3.1. Definitions

**Figure 2:**
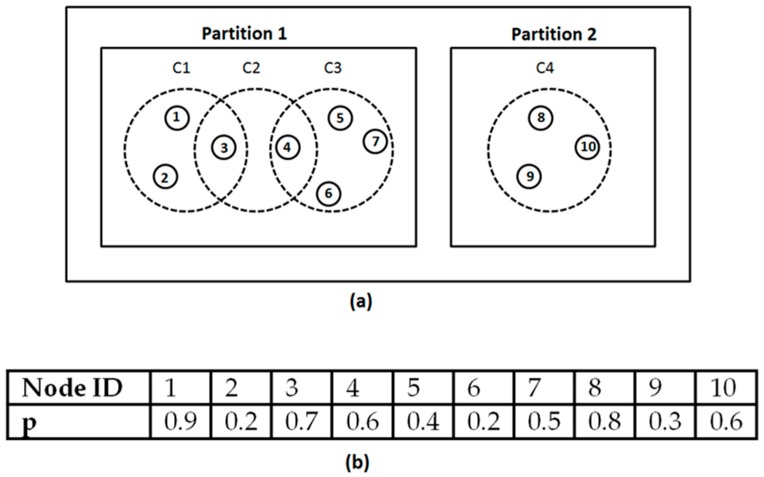
(**a**) Difference between cluster and partition, (**b**) Example values of *p*.

Cluster: a cluster is a group of unlocalized nodes in which every node is within communication range of every other node. Assuming in Figure 2a that nodes 1, 2, and 3 are within each other’s communication range, they form cluster C1. Similarly, nodes 3 and 4 form cluster C2. Nodes 4, 5, 6, and 7 form cluster C3, and nodes 8, 9, and 10 form cluster C4, respectively. It is important to note that the size of a cluster depends on the current communication range. Therefore, as we increase the transmission power every iteration, the cluster size may also increase as more nodes fall within each other’s range. The increase in cluster size will decrease the number of clusters.

Cluster Head: A cluster head (CH) is a representative node that is responsible for sending localization requests on behalf of a cluster. On reception of a localization beacon in response to its localization request, the cluster head localizes itself. Moreover, it creates an updated beacon by adding its newly calculated absolute coordinates and transmits the updated beacon. As all the nodes in a cluster are within CH’s communication range, all the nodes in the cluster will receive the updated beacon. These nodes will use the beacon to estimate their absolute coordinates. A CH is chosen based on a random value *p* added to every localization request. The node with the highest *p* within a cluster becomes the cluster head.

Partition: A partition refers to a group of nodes in which a packet, once transmitted by any node X, will eventually be received by all the nodes in that group, either directly from node X, or routed through other ordinary sensor nodes. A partition may consist of one or more overlapping clusters. Figure 2a shows two partitions: partition 1 and partition 2. Partition 1 consists of three overlapping clusters C1, C2, and C3, whereas partition 2 consists of a single cluster C4. In the network shown in Figure 2a, a packet transmitted by any node in partition 1 will eventually reach all the other nodes in partition 1. However, the packet will not reach any of the nodes in partition 2, as none of the nodes in partition 2 is in the communication range of any of the nodes in partition 1. For example, a packet transmitted by node 1 will reach node 7 routed through nodes 3 and 4, but it will not reach any of the nodes in partition 2. Thus we have two partitions, partition 1 and partition 2. 

Based on the values of *p*, it is possible that a partition may have only one cluster head, irrespective of the number of clusters. For the values of *p* shown in Figure 2b, only node 1 will be selected as the cluster head for all the nodes in partition 1. This can be explained as follows: Node 4 has the highest value of *p* in C3, therefore the other nodes in C3 assume node 4 as CH. However, in C2, node 3 has a higher value of *p* than node 4; therefore, instead of assuming itself as CH, node 4 assumes that node 3 is cluster head, whereas node 3 assumes node 1 as CH. Thus, node 1 becomes the only representative node for the whole partition, and requests beacon on behalf of the whole partition. As all nodes in a partition are directly or indirectly connected, the beacon received by the cluster head (i.e. node 1) will eventually reach every node in partition 1.

**Figure 3:**
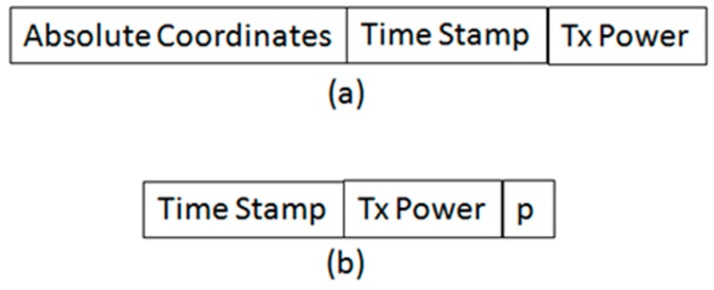
(**a**) Format of Localization Beacon, and (**b**) Format of localization Request.

Format of beacon: Figure 3a shows the format of a beacon. The absolute coordinates are the GPS coordinates of the node that is transmitting the beacon. The time stamp contains the time of transmission of the beacon. This time is used by the receivers to estimate their position relative to the sender using TDoA. (It is important to note that we assume every node is equipped with three non-collinear antennae for TDoA estimation). Tx Power is used by the receiver to estimate the transmission range of the sender, which is used for the retransmission control mechanism that is explained in Section 3.2.3.

Format of localization request: Figure 3b shows the format of a localization request. TX Power along with the received power and time stamp is used by the GPS node to estimate the distance of the sender. The GPS node adjust its transmission power according to the calculated distance in order to ensure that the sender receives the localization beacon that it will send in response to the senders localization request. *p* is a random decimal number between zero and 1. It is used by the receiving unlocalized nodes to determine whether they should become a cluster head, or a cluster member. The time stamp is the time of transmission of the localization request.

### 3.2. The Localization Process

Our proposed localization procedure is carried out periodically every *T* units of time. Every instance of the localization process is called a localization cycle, which consists of two phases: (a) the reactive phase, during which all single and multi-hop neighbors of the GPS node are localized, and (b) an iterative proactive phase, during which the partitioned nodes (e.g., the nodes that could not receive beacon during phase 1) are localized. 

Our proposed localization procedure is carried out periodically every *T* units of time. Every instance of the localization process is called a localization cycle, which consists of two phases. In the first phase, the GPS-enabled node initiates the localization. During this phase, all the single and multihop neighbors of the GPS node are localized. However, partitioned nodes, i.e. the nodes that are outside the communication ranges of the GPS node and all of the localized nodes, will not be able to receive the beacon during phase 1. All these unlocalized nodes wait for a certain predetermined threshold time *T_p2_*. After *T_p2_*, they proactively request beacons from the GPS node. Phase 2 is an iterative phase. Each iteration of phase 2 is defined by the following steps:

(1) Transmission of localization requests with doubled transmission range (In this work we use Evo Logic’s S2CR 48/47 acoustic modem in which transmission range is doubled every next available level of transmission power. Moreover the formulation in Section 4.1 can also be used to calculate the transmission power required to achieve a certain communication range.): At the start of every iteration, all unlocalized nodes or their representative nodes send localization requests with doubled transmission range. If a beacon is not received in response to a localization request, the localization request is sent in the next iteration with doubled transmission range.
(2) The selection of cluster heads for the next iteration: Clusterheads (CH) are selected during each iteration based on random values *p* contained in every localization request. A CH selected during a particular iteration sends localization requests in the next iteration.
(3) Reception of localization beacon in response to localization request, and calculation of GPS coordinates using the beacon.
(4) Every freshly localized node runs a “retransmission control” mechanism to decide on the retransmission of an updated beacon.

We assume a network of freely mobile underwater sensor nodes that are equipped with the necessary apparatus for TDoA calculation, i.e. every node hosts three non-collinearly positioned antennae. All nodes are time synchronized, and transmit with the same default transmission range (*R_d_*) that yields the transmission range R. The transmission power can be increased or decreased, if a change in transmission range is required. However, during a particular iteration, all the nodes transmit with the same transmission power, where *i* denotes the iteration number.

#### 3.2.1. Phase 1: The Reactive Stage

Phase 1 is initiated when the GPS-enabled node floods a beacon into the network over default transmission range. This triggers a process during which the receiving nodes: (a) estimate their GPS coordinates using the received beacon; (b) save the relative coordinates of the sender to be used for retransmission control (Section 3.2.3); (c) create updated beacons containing their newly estimated coordinates, and (d) transmit the updated beacons, which are used by the unlocalized nodes that receive them to estimate their absolute coordinates. All the nodes that receive the updated beacons follow the same steps. During this phase, all single and multi-hop neighbors of the GPS node are localized. However, the nodes that are partitioned due to free mobility (i.e. the nodes that are out of the default communication ranges of the GPS node and all of the localized ordinary nodes) will not be able to receive the beacon in phase 1, and therefore will remain unlocalized. These nodes are localized in phase 2. All these unlocalized nodes wait for a certain predetermined threshold time *T_p2_*. After *T_p2_*, they proactively request beacons from the GPS node by sending localization requests. *T_p2_* is counted from the start of phase 1. Every node can calculate *T_p2_*, as all the nodes are time synchronized, and the starting time of all the localization processes is known in advance.

#### 3.2.2. Phase 2: The Proactive stage

Phase 2 is an iterative phase. Every iteration of phase 2 is defined by the following steps. 

##### Transmission of Localization Requests with Doubled Transmission Range

At the start of every iteration, all unlocalized partitioned nodes (iteration 1), or their representative nodes, i.e. cluster heads (iteration 2 and onwards) send localization requests with doubled transmission range. (Transmission range is doubled every iteration. However, during a particular iteration, all transmitting nodes use the same transmission range to transmit localization requests.) A localization request serves as a request to the GPS node to send a localization beacon. If the GPS node is within the increased transmission range of a sender, it responds to the localization requests by sending a beacon. However, if the GPS node is not within the increased transmission range of a sender, it will not receive the localization request, and therefore will not send a beacon. This may result in a situation where some nodes (whose localization requests could not reach the GPS node) are left unlocalized. In such a case, the CHs selected during the current iteration will send localization requests over a doubled transmission range in the next iteration in order to reach the GPS node (the process of cluster head selection is explained in the following section). 

The duration of any iteration *i* (Equation (1)) is equal to the sum of the processing delay (*D_pr_*) and thrice the maximum propagation delay, i.e. *R/C*, where *R* is the current range, and *C* is the speed of sound under water. This ensures that if the GPS node is within the range of a particular transmitting node, the node must receive the beacon before the end of the current iteration. It also ensures that if a receiver retransmits the beacon, it reaches every node within its range before the end of the current iteration.

(1) Iduration=3RC+Dpr

##### Clustering and Cluster Head Selection

As explained earlier, a cluster head is a representative node that sends localization requests on behalf of the unlocalized nodes within its cluster, or within the partition of which it is a part. Only unlocalized nodes participate in the CH selection process, which is explained as follows:

Localization requests transmitted at the start of any iteration *i* are received by all the nodes within the range of the senders. Every localization request contains a value *p* randomly chosen by the sender of the request (In a particular iteration *i*, all transmitting nodes transmit over equal transmission range. Therefore if a node A can receive a localization request transmitted by another node B, then node B can also receive the localization request transmitted by A. Therefore, each of the two nodes knows the value *p* chosen by the other node, and can compare that value with its own to decide when it should be a CH or a Cluster Member (CM).). If a node that sent a localization request receives a localization request sent by another node within *T* (Equation (2)) seconds of its own transmission, it compares the random value *p* in to the received localization request with its own. At the end of time period *T*, a node x will become a cluster head (CH) if the value *p* chosen by node x is higher than all the values of *p* that it received. In such a case, node x will send localization requests on behalf of the whole cluster in the next iteration, i.e. iteration *i*+1, if the cluster remains unlocalized during the current iteration. However, if node x’s value of *p* is smaller than any of the received values, it becomes a cluster member, and relieves itself from sending localization requests.

(2) T=R/C

where *R*, is the range during iteration *i* (all transmitting nodes have equal transmission range during any iteration) and *C* = 1500 m/s is the propagation speed of sound in water. As all nodes within a cluster are within the range of each other, a localization request transmitted by any node in the cluster should reach every node in the cluster within *T* seconds.

##### Reception of Localization Beacon and Retransmission of an Updated Localization Beacon

As explained earlier, a localization request serves as a request for a beacon from a GPS node. If a node receives a localization beacon in response to its localization request (beacon is received either directly from the GPS node in response to a localization request, or it can be an updated beacon transmitted by another ordinary sensor node that recently localized itself), it localizes itself using the beacon. Moreover, if required the node updates the beacon with its own absolute coordinates and retransmits the updated beacon with its last used transmission range. Clusters are defined based on the communication range as defined in Section 3.1. Therefore, the retransmission of a beacon with the last used transmission range ensures that all the nodes within the transmitter’s range receive the beacon (Section 3.2.3 explains when a node is required to retransmit a fresh beacon in phase 2). A beacon is retransmitted to ensure that all the unlocalized nodes in the transmission range of the sender also receive the beacon. The retransmission of beacons by every freshly localized node also ensures that if even a single node in a cluster (or partition) receives a beacon, the beacon will eventually be received by all the nodes in the cluster (or partition), thus localizing all the nodes. This will eliminate the need to send further localization requests.

If a localization beacon is not received within time *T_response_* (Equation (3)) after the transmission of the localization request, it is assumed that the localization request could not reach the GPS node. In such a case, the clusterhead(s) selected during the current iteration waits until the end of the current iteration and initiates the next iteration by transmitting a localization request over double the current transmission range. This process continues, until all the nodes are localized, or the maximum number of iterations is reached.

(3) Tresponse=2RC+DPr

where, *R* is the current range of the sensor nodes, *C* is the speed of sound under water, and *D_pr_* is the processing delay.

##### Cluster Merging

As the transmission range is doubled every iteration, the cluster heads representing different clusters may fall within one another’s range. This will result in the clusterheads receiving the localization requests transmitted by the other CHs within their communication range. Based on the values of *p* in the received localization requests, some clusterheads (i.e. those having smaller values of *p*) will become cluster members in the extended cluster, whereas those with the highest value among all the CHs within their range will become CHs for the next iteration. We may call these CHs clusterheads of clusterheads. This forms a hierarchical structure, in which nodes higher up the hierarchy are responsible for nodes immediately below them in the hierarchy (further explained in the example scenario in Section 3.2.4).

#### 3.2.3. Retransmission Control: Decision Making on the Retransmission of a Received Beacon

In this work, we assume that every node that receives a packet from another node calculates the position of the sender relative to itself and saves it in a table of relative positions. 

The tables maintained by nodes are used for a retransmission control mechanism. For instance, when node y sends a localization request destined to the GPS node, node x may also hear the transmission if it is located within the transmission range of node y. In such a case node x estimates and saves the relative position of y relative to itself. This information can be used in the retransmission control process.

**Lemma** **1.** Assume three nodes A, B, and C. B can estimate the distance of C from A along the X-axis and Y-axis using Equations (4) and (5), respectively, if it knows the positions of A and C relative to itself.

In Equations (4) and (5), *X_ij_* and *Y_ij_* stand for the relative positions of node j relative to node *i* along the *X*-axis and *Y*-axis, respectively. Whereas, *DX_ij_* and *DY_ij_* represent the distance of node *i* from node *j* along the *X*-axis and *Y*-axis, respectively.

(4) $$DX_{ca}=abs(X_{cb}-X_{ab})$$

(5) $$DY_{ca}=abs\left(Y_{cb}-Y_{ab}\right)$$

Then using *DX_ca_* and *DY_ca_*, and assuming one of the two nodes on *x* = 0 and *y* = 0, node B can estimate the distance D between nodes A and C using the two-point distance formula (Equation (6)):

(6) $$D=\sqrt{{\left(DX_{ca}-0\right)}^{2}+{\left(DY_{ca}-0\right)}^{2}}$$

Any node B that receives a beacon from another node A can use the TX power information contained in the beacon to calculate the transmission range of node A. Using the transmission range of A along with its table of relative positions and distance *D* between node A and C (calculated using Equation (6)), node B can decide whether it needs to retransmit an updated beacon or not. If all the neighbors of node B are within the communication range of node A, then node B will not retransmit the beacon, as its neighbor also receives the beacon transmitted by node A. However, if any of the neighbors of node B (say node C) is out of node A’s communication range, node B will retransmit the beacon, so that node C can localize itself. 

#### 3.2.4. Example Scenario 

**Figure 4:**
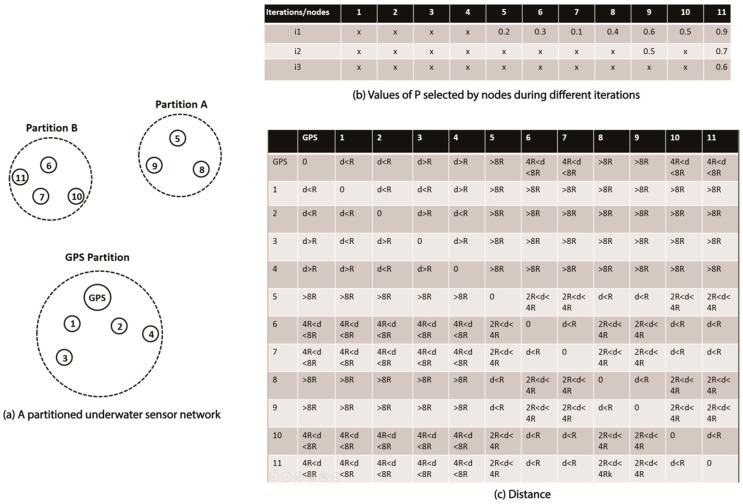
(**a**) Partitioned underwater sensor networks, (**b**) Values of *p*, and (**c**) Distances between nodes.

Our scheme can be explained with the help of Figure 4, which shows an underwater sensor network in which nodes have drifted away into three partitions: the GPS partition, partition A, and partition B. For the sake of simplicity, we assume that every node in a particular non-gps partition (i.e. partitions A and B) is within the range of all the other nodes in that partition. Whereas in the GPS partition, node 1 and node 2 are within the default range of the GPS node. Node 3 and node 4 are within the range of node 1 and node 2, respectively. However, node 3 and node 4 are outside each other’s range, and also that of the GPS node. 

##### Stage 1: The Reactive Stage

**Figure 5:**
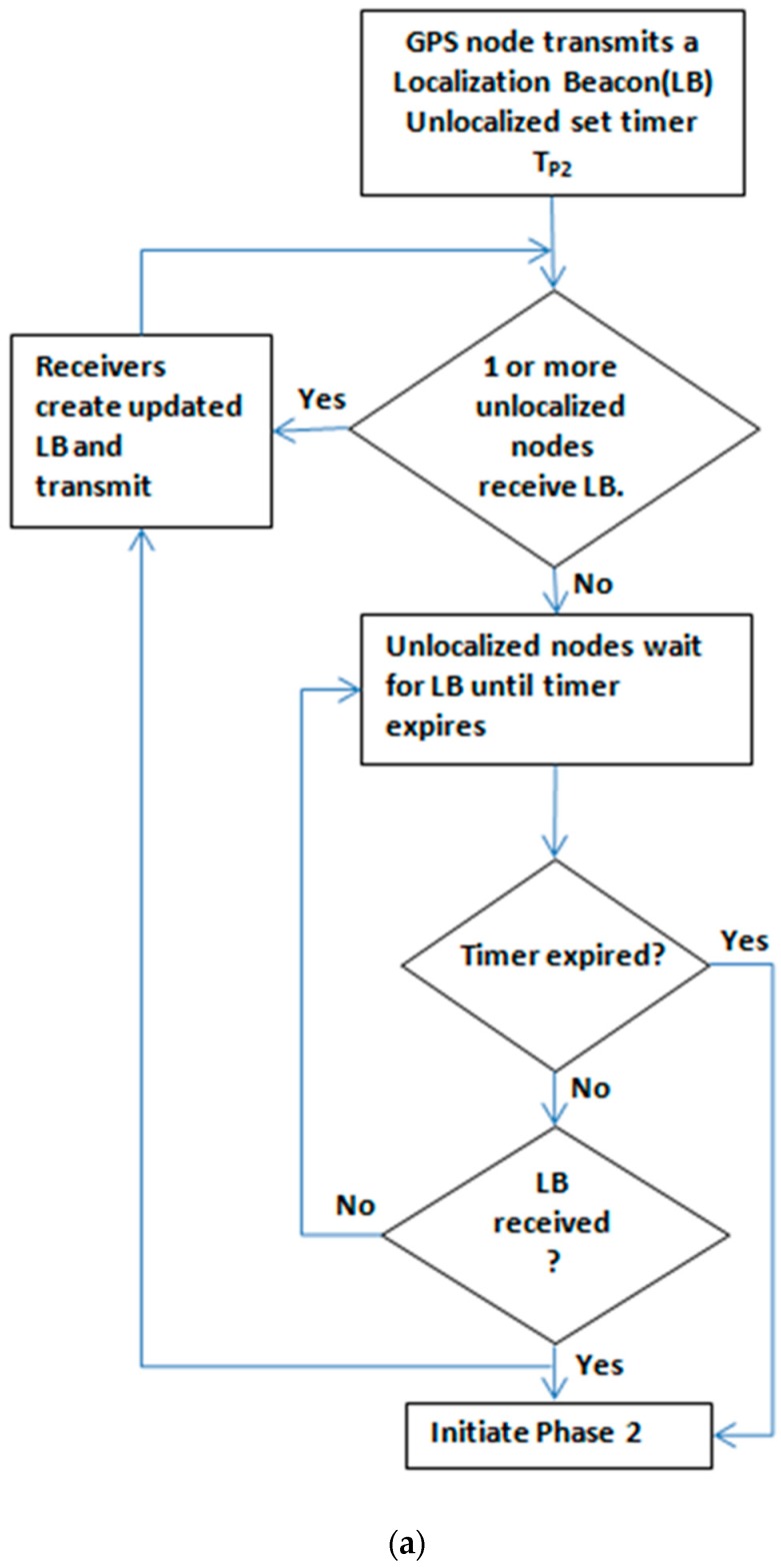

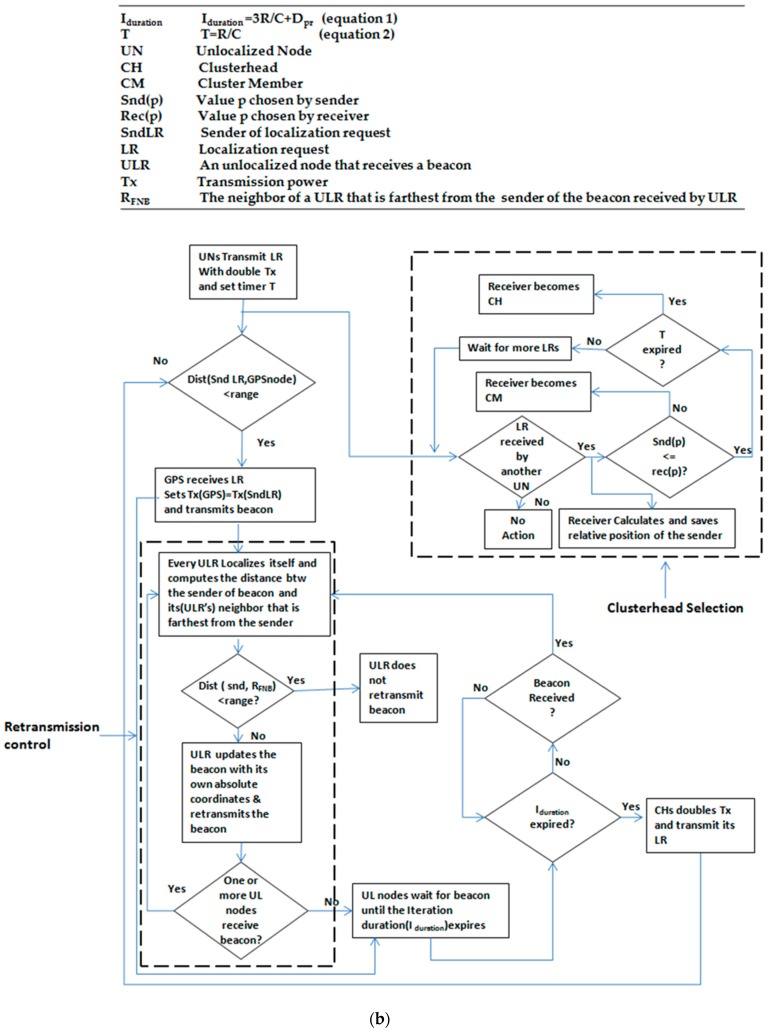
(**a**) Flow chart Phase 1; (**b**) Flow chart Phase 2.

The GPS node initiates the localization cycle at a pre-scheduled time t by transmitting a beacon. The beacon is received by node 1 and node 2. The two receiving nodes estimate their absolute coordinates using the beacon. Each of them transmits a fresh beacon with its own newly estimated coordinates. The beacon transmitted by node 1 is heard by the GPS node, node 2, and node 3. Similarly, the beacon transmitted by node 2 is heard by the GPS node, node 1, and node 4. As the GPS node and node 2 (in the former case), and the GPS node and node 1 (in the latter case) have already been localized, they drop the beacon. However, node 3 and node 4 will accept the beacons transmitted by node 1 and node 2, respectively. These nodes follow the same steps as their predecessors, i.e. they estimate their GPS coordinates using the received beacons and transmit the updated beacon with their own absolute coordinates. There is no unlocalized node within the range of nodes 3 and 4, and therefore, the beacon propagation stops there. (Please refer to Figure 5a to see the flow chart diagram of stage 1). As a result, nodes 5 to 11 will not receive any beacon, and remain unlocalized. However, all these nodes wait for a certain predetermined threshold time *T_p2_*. After *T_p2_*, they proactively request beacons from the GPS node by sending localization requests. We call this phase, phase 2, or the proactive stage.

##### Stage 2: The Proactive Stage

The proactive stage or stage 2 can be explained using Figure 4 and the flow chart diagram in Figure 5b.

At the end of threshold time *T_p2_*, all the unlocalized nodes initiate phase 2. During the first iteration of phase 2, all unlocalized nodes transmit localization requests with double the default transmission range. As all the three nodes in partition A, i.e. nodes 5, 8, and 9, are within each other’s transmission range (Figure 4c), each of them receives localization requests transmitted by the other two. After comparing the values of *p* (Figure 4b), node 9 assumes the role of clusterhead, as it has the highest value of *p*, while nodes 8 and 5 find out that they have *p* smaller than that of node 1, and therefore they go into reactive mode, i.e. they relieve themselves of the responsibility of sending localization requests from the next iteration onwards.

A similar process occurs in the case of nodes 6, 7, and 10, where node 11 is selected as CH, because it has the highest value of *p*. It is important to note that the two sets of nodes are not accessible to each other, thus making two partitions. Every partition has its own CH, which will send localization requests with a higher transmission range in the next iteration, if the partition remains unlocalized during the current iteration. 

Cluster Merging: Consider the two clusters, i.e. partition A and partition B *(here, the clusters and partitions are the same) shown in Figure 4a. We know that node 9 and node 11 have been selected as CHs for their respective clusters during iteration 1. As with the current range, i.e. 2R, the localization request from any of the nodes in partition A and partition B would not reach the GPS node (see Figure 4c), and none of the nodes in the two clusters will receive any beacon during iteration 1. Therefore, both CHs, i.e. nodes 9 and 11, will start iteration 2 by transmitting localization requests with a transmission range equal to the double of the previous iteration, i.e. 4R. As nodes 9 and 11 are within the distance of 4R from each other, they receive each other’s localization requests. Therefore, based on the values of *p* (see iteration 2 (i2) in Figure 4b) in their localization requests, one of them (node 11 in our case) is selected as CH, where node 9 is relieved of its responsibility of sending localization requests from the next iteration (iteration 3) onward. This will merge the two clusters, i.e. partition A and partition B, as now only node 11 will send Localization Requests (with double the previous transmission range) on behalf of both the clusters, if a beacon is not received in the current iteration. The distance of the GPS node from both nodes 11 and 9 is higher than 4R (the current transmission range). Therefore, neither of the two nodes, i.e. neither 9 nor 11, will receive a beacon in response to their localization requests. Node 11 will start iteration 3 by transmitting a localization request with double the current transmission range, i.e. 8R. As the GPS node is within the new range of node 11, it will receive the localization request, and respond with a beacon using TX equal to 8R, thus making sure that it reaches node 11. As we know that a node retransmits a beacon with its last used range, therefore when node 11 receives a beacon, it will retransmit it with 8R. As node 9 is within the distance of 8R from 11, it will receive the beacon, and will retransmit it for its own cluster with its last used range, i.e. 4R. This will ensure that all the nodes in partition A receive the beacon. 

Figure 4b shows the values of *p* selected by nodes during different iterations. It is important to note that the figure shows values of *p* for all the nodes for the first iteration. However, this figure shows values of *p* for only nodes 11 and 9 during the second iteration, and only for node 11 during the third iteration. This can be explained as follows. During the first iteration, every unlocalized node transmitted a localization request. Based on the values of *p* in these localization requests (as shown in Figure 4b), node 11 and node 9 were selected as cluster heads. During the second iteration, only the cluster heads, i.e. node 11 and node 9, transmitted localization requests on behalf of their clusters with increased transmission power. Due to the increase in transmission range, nodes 11 and 9 both receive the localization request transmitted by the other. Based on the values of *p* in their localization requests (Figure 4b), node 11 is selected as the cluster head. From this point on, only node 11 sends localization requests, doubling TX range every iteration, until a beacon is received in response to its localization request.

##### Using TDoA for Localization of Sensor Nodes 

**Figure 6:**
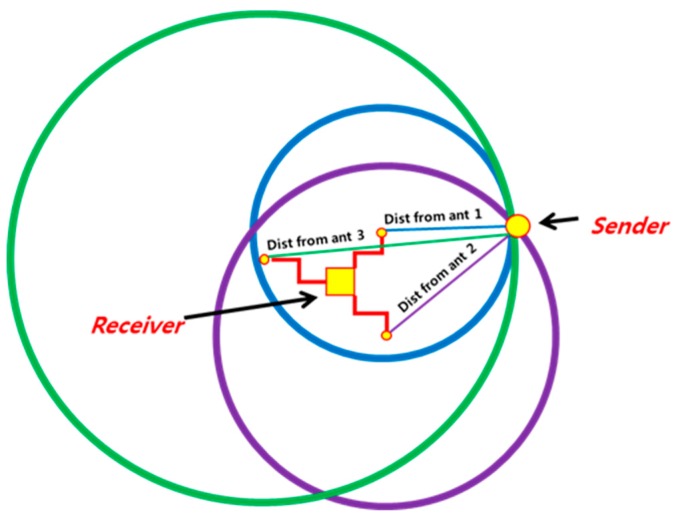
Use of TDoA for localization.

Figure 6 illustrates use of TDoA for localization of sensor nodes. Every node is equipped with three non-collinearly located antennae. The arriving signal is received by the three antennae, each of which estimates its distance from the sender. This information is centrally processed by the receiving node which applies trilateration to estimate relative position of the sender.

## 4. Simulation Setup

### 4.1. Energy Consumption Model of Underwater Acoustic Channel

The attenuation of acoustic signals in an underwater acoustic channel is widely modeled as Equation (7) [17], where *d* represents the transmission range, *f* represents the signal frequency, and *S* represents the spreading loss. The commonly used values of *S* are (1, 2, and 1.5) for cylindrical, spherical, and so-called practical spreading, respectively. *α*(*f*) is the absorption coefficient. Equation (8) [17] estimates the value of *α*(*f*) in dB/km, when the frequency is measured in kHz.

(7) $$\gamma \left(d,f\right)=d^{s}*\propto {\left(f\right)}^{d}$$

(8) $$10\;log\propto \left(f\right)=0.11\frac{f^{2}}{1+f^{2}}+44\frac{f^{2}}{4100+f^{2}}+2.75\times 10^{-4}f^{2}+0.003$$

Equation (8) is generally valid when the frequency *f* is above a few hundred Hz. However for lower frequencies, it is suggested to use Equation (9) [17].

(9) $$10log\alpha \left(f\right)=0.002+0.11\frac{f^{2}}{1+f^{2}}+0.011f^{2}$$

The power consumption for transmitting a packet over the range of *d* meters and frequency *f* in khz can be estimated using Equation (10) [17]:

(10) $$P_{db}\left(d,f\right)=\eta \left(f\right)\gamma \left(d,f\right)\beta \left(f\right)SNR$$

where, η(*f*) represents power spectral density, β(*f*) represents usable bandwidth around the center frequency *f*, and *SNR* represents the target signal-to-noise ratio at the receiver, respectively. 

The conversion from acoustic power $P_{db}\left(d,f\right)$ in dB to electrical power $P_{watts}\left(d\right)$ in watts is given as follows by Equation (11) [18]:

(11) $$P_{watts}\left(d\right)=P_{db}\left(d,f\right)*10^{-17.2}/\sigma$$

where, $10^{-17.2}$ is the conversion factor, and σ is the overall efficiency of the electric circuitry.

The total power consumption is calculated using Equation (12) [19]:

(12) $$TP=P_{watts}\left(d\right)+P_{R}$$

where, $TP,\;P_{watts}\left(d\right),\;\mathrm{and}\;P_{R}$ are the total power for communication over a distance *d*, transmission power, and the fixed overhead for receiving data, respectively. All of these quantities are measured in watts. 

The total energy consumption for single transmission of a packet of size *X* bits transmitted with a transmission rate ρ is given by Equation (13):

(13) $$\varepsilon =TP*\frac{X}{\rho }$$

### 4.2. Parameters Setting

We used Matlab R2016b to simulate our protocol. Table 1 shows the simulation parameters. Nodes are assumed to move freely with currents. However, we also assume that during a localization period, their positions relative to each other do not change. This assumption is reasonable, as all the nodes move within the same current. The node distribution in the network is as follows.

1. Nodes are distributed over the simulation area using normal distribution.
2. Nodes are distributed in the simulation area in such a way that they form 2 partitions.
3. Nodes are distributed in the simulation area in such a way that they form 3 partitions.
4. Nodes are distributed in the simulation area in such a way that they form 4 partitions.

Simulation results are averaged over the above given four cases of node deployment for 100 simulation runs each. 

For AUV aided localization, the AUV follows a spiral path. The total length of the spiral path is roughly 5000 m. The AUV transmits a beacon every 50 m. Node deployment for AUV aided localization is the same as above. 

## 5. Results and Discussion

In this section, we compare our technique (referred to as 2SC) with the technique used by TP-TFSLA during its stage 2 to localize partitioned nodes. We refer to this technique as a simple two-stage localization without clustering, or 2S.

### 5.1. Communication Overhead

Communication overhead C given by Equation (14) is the ratio of the total number of messages exchanged to the total number of localized nodes:

(14) $$C=\eta_{T}/\eta_{L}$$

where, $\eta_{L}$ is the total number of localized nodes, and $\eta_{T}$ is the total number of transmissions to carry out the localization.

**Figure 7:**
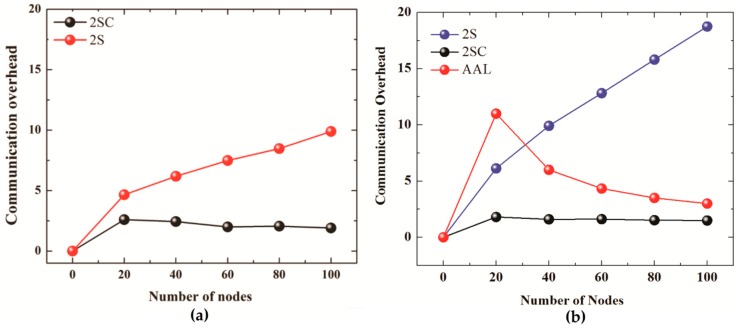
(**a**) Communication overhead for No. of iterations = 2. (**b**) Communication overhead for No. of iterations = 3 (2S, 2SC), AUV Aided Localization.

Figure 7a shows the communication overheads of 2SC and 2S for the number of iterations set to 2 whereas Figure 7b compares the communication overheads of 2SC and 2S (number of iterations set to 3) with the communication overheads of the AUV aided localization scheme.

The number of nodes is set to 20, 40, 60, 80, and 100. In 2S, the communication overhead increases from almost 5 for 20 nodes to 10 for 100 nodes in the case of 2 iterations, and from 6 for 20 nodes to almost 18 for 100 nodes in the case of 3 iterations. However, in the case of 2SC, the communication overhead stays low at around 2. The slight decrease in the communication overhead of 2SC with the increase in number of nodes is due retransmission control, which becomes more effective in denser networks due to the close proximity of nodes. The communication overhead of AUV aided localization in Figure 7b is high for 20 nodes but decreases with an increase in the number of nodes. This is due to a fixed overhead incurred by the periodic transmission of beacons every 50 m as the node traverses a spiral path in the network.

The reason for the low communication overhead in the case of 2SC is the clustering technique that selects one clusterhead per cluster (or per partition). The cluster head sends localization requests on behalf of the whole cluster (or partition). Moreover, as the transmission range is doubled every iteration, the clusters tend to merge. The merger decreases the number of clusterheads, thus reducing the number of transmissions even more. Moreover, a significant reduction in the communication overhead is also achieved through the “retransmission control” technique discussed in Section 3.2.3. This technique ensures that unnecessary transmissions are stopped, thus reducing communication overheads. Another reason for the low communication overhead is that in 2SC, only the GPS node responds to a localization request, thus reducing the number of beacons to a much smaller number compared to 2S, where all those localized nodes that receive a beacon attempt to respond with a beacon. 

The slight decrease in the communication overhead of 2SC in case of 3 iterations compared to 2 iterations is due to the following reason. As the number of iterations increases, the clusters tend to merge. This results in reducing the number of clusterheads and thus the number of transmissions. Moreover, due to an increase in the communication range as the number of iterations increases to 3, retransmission control also becomes more effective. This results in a reduction of communication overheads.

### 5.2. Energy Consumption

We assume that all nodes are equipped with Evo Logic’s S2CR 48/47 Acoustic modem. Table 2 shows power consumption by the modem for transmitting over different ranges [21]. For ranges above 1000 m we use 35W instead of 60W. The reason for this is that S2CR 48/47 acoustic modem has a max range of 2000 m which is catered for by 60 W. However, in our case the max range should not be more than 1200 m, which, we assume, can be achieved using 35W. 

**Figure 8:**
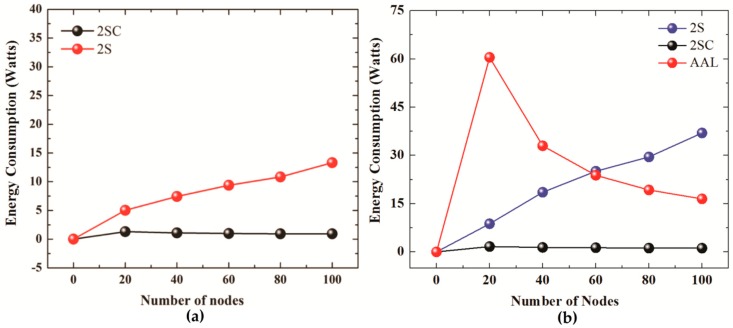
(**a**) Energy Consumption for No. of iterations = 2. (**b**) Energy Efficiency for No. of iterations = 3 (2S, 2SC), AUV Aided Localization.

Figure 8a shows the energy consumption results of 2SC and 2S for the number of iterations set to 2 whereas Figure 8b compares Energy consumption of 2SC and 2S (number of iteration set to 3) with the energy consumption of AUV aided localization scheme. The Energy consumption is computed using Equation (15):

(15) $$Є=\frac{T_{EC}}{N}$$

where, Є represents the average energy consumption, and $T_{EC}$ represents the total energy consumed by the localization procedure when the total number of nodes is *N*.

The figures suggest, 2SC uses much a smaller amount of energy, compared to 2S. As the number of nodes increases from 20 to 100, 2SC shows better energy efficiency compared to 2S. This, besides better energy consumption, also points to the better scalability of 2SC to networks with a larger number of nodes. The reason for smaller energy consumption in the case of 2SC is a smaller communication overhead as a result of clustering, retransmission control, and restricting localized ordinary sensor nodes from responding to a localization request. 

Comparison of the energy consumption graphs for the number of iterations equal to 2 (Figure 8a) and number of iterations equal to 3 (Figure 8b) reveals that an increase in the number of iterations (which essentially means that all the nodes could not be localized in 2 iterations) widens the gap in the energy consumption performance of the two protocols. This is due to the fact that in 2SC, clusters tend to merge, due to doubling of transmission range every iteration. The merging of clusters decreases the number of clusterheads, thus fewer transmissions are made compared to the previous iteration. Moreover, an increase in the transmission range also results in the reduction of retransmissions due to the application of “retransmission control”. This results in a sharp decrease in the number of transmissions required to localize nodes in that particular iteration. In other words, in 2SC, the communication overhead, and therefore energy efficiency, in a particular iteration decreases for every next iteration, resulting in widening the gap between the two curves at every next iteration. This points to the better scalability of our protocol. AUV aided localization involves higher energy consumption due to the big number of beacon transmissions by the AUV as it traverses the network in a spiral motion. The AUV transmits a beacon every 50 m. As the number of beacon transmissions remains almost the same, the overall energy consumption for a smaller number of nodes is higher.

### 5.3. Localization Error

Common causes of localization errors include errors in the speed of sound and depth measurements, time stamping errors, underwater multipath fading and projection accuracy [20]. 

**Figure 9:**
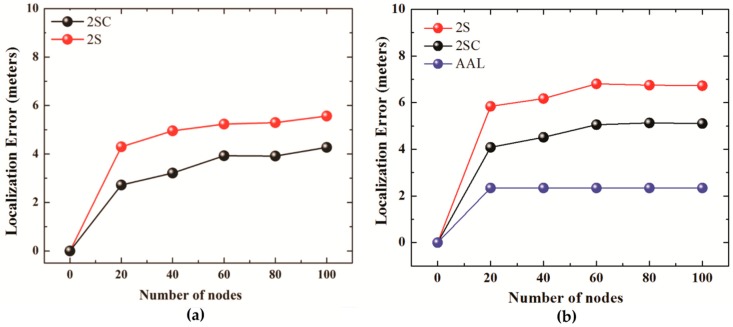
(**a**) Localization error for No. of iterations = 2. (**b**) Localization error for No. of iterations= 3(2S, 2SC), AUV Aided Localization.

Figure 9a shows the localization error of 2SC and 2S for the number of iterations set to 2 whereas Figure 9b compares localization errors of 2SC and 2S (number of iteration set to 3) with the localization error of AUV aided localization scheme.

The results show that the average localization error in 2SC is smaller, compared to that in 2S. The reason for a smaller localization error in the case of 2SC lies in allowing only the GPS node to send localization beacons. This results in avoiding error accumulation that occurs in the case of 2S, in which already localized nodes send their own coordinates (derived using the coordinates of the GPS node or other localized node) in response to localization requests.

We also notice that as the number of iterations increases from 2 to 3, the localization error experienced by 2SC, as well as 2S, increases. The small increase in the case of 2SC is due to the increase in distance related errors, and also due to the increase in hop count. The hop count in the case of 2SC may increase with iterations, due to the merging of clusters. As the clusters merge, a hierarchal structure is created, in which a CH may become representative of other CHs, which in turn are representatives of other CHs and/or nodes, thus increasing in the number of hops. In the case of 2S, as more nodes are localized every iteration, the number of hops between the GPS node and the unlocalized node requesting beacons increases, thus increasing error accumulation. As oppose to 2SC and 2S, the AUV aided localization scheme does not have error accumulation as sensor nodes receive beacons directly from the AUV. This also results in a very small variation in localization error as error accumulation is avoided.

### 5.4. Localization Coverage

**Figure 10:**
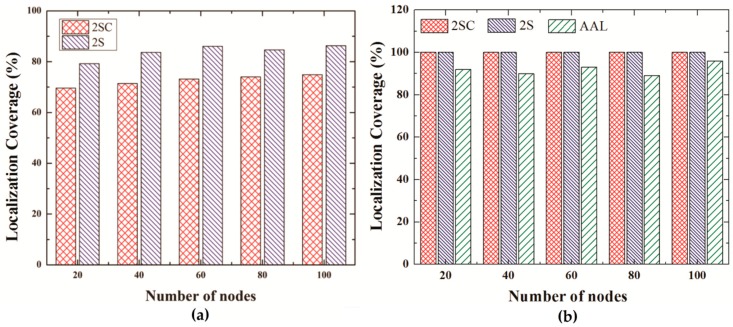
(**a**) Localization Coverage for No. of iterations = 2. (**b**) Localization Coverage for No. of iterations = 3(2S, 2SC), AUV Aided Localization.

Figure 10a shows the localization coverage of 2SC and 2S for the number of iterations set to 2 whereas Figure 10b compares localization coverage of 2SC and 2S (number of iteration set to 3) with the localization coverage of AUV aided localization scheme.

When the number of iterations is 2, 2S shows slightly better performance than 2SC. However when the number of iterations increases, both protocols can propagate beacons to all the nodes, thus resulting in the localization of all the nodes. The better localization coverage of 2S is due to the fact that in 2S, any localized node can respond to a localization request with a beacon. However, in the case of 2SC, only the GPS node responds to localization requests, thus increasing the chances that a localization request may not reach the GPS node, and therefore may not receive a beacon to localize itself in the current iteration. In the case of AUV aided localization, the localization coverage is low despite of low error. This is because the AUV follows a spiral path and therefore some of the nodes located on the edges of the network may not receive three beacon.

### 5.5. Effect of CH Position

**Figure 11:**
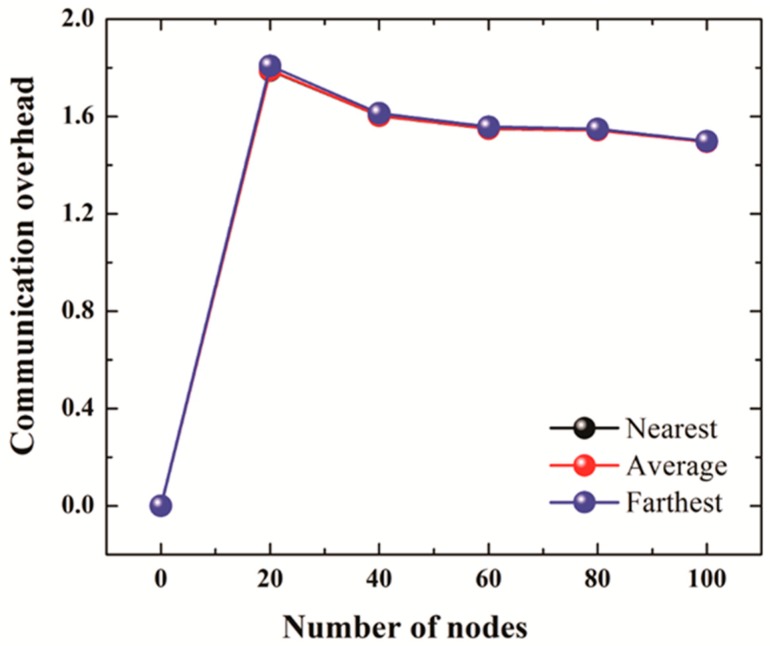
Communication overhead for different CH positions.

**Figure 12:**
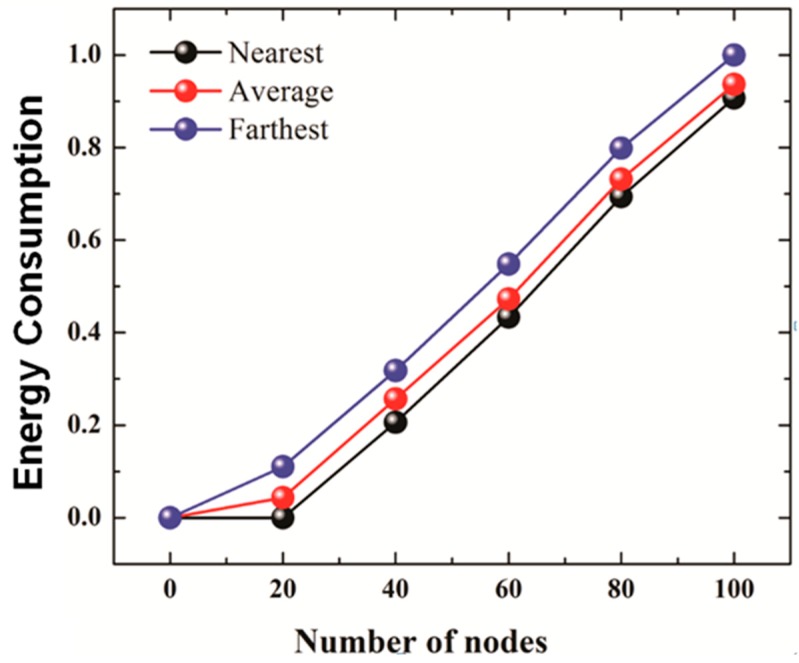
Energy Consumption for different CH positions.

**Figure 13:**
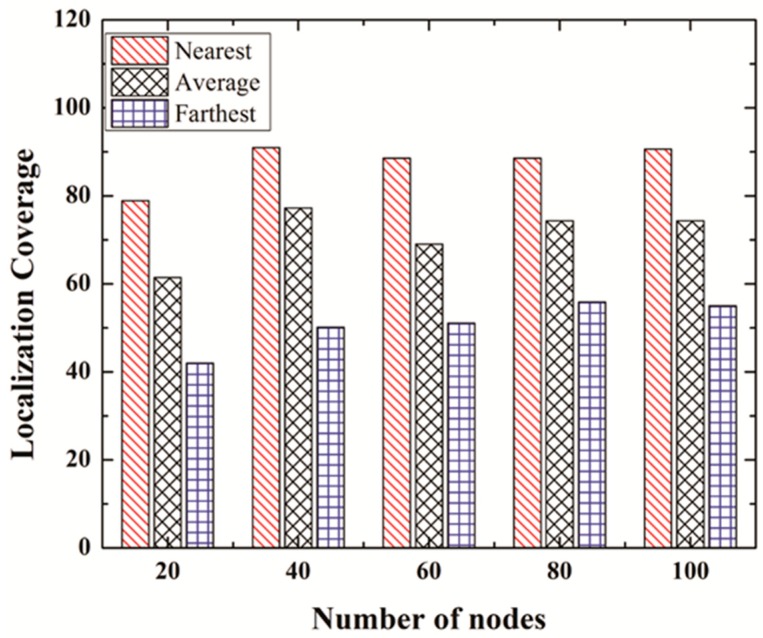
Localization coverage for different CH positions.

CH is selected based on value *p* chosen randomly by every transmitting node. Therefore, any node in the cluster can become a CH, if its selected value *p* is the highest among the other nodes in the cluster. In this section we evaluate how the distance of a CH from the GPS node affects communication overhead (Figure 11), energy consumption (Figure 12), and localization coverage (Figure 13). We consider 3 cases:

1. Nearest Case: CH is the nearest node to the GPS node among all the nodes in a cluster.
2. Average Case: CH is selected based on value *p*.
3. Farthest Case: CH is the farthest node to the GPS node among all the nodes in a cluster.

#### 5.5.1. Communication Overhead

Communication overhead stays almost similar, irrespective of the position of the CH. This is because only clusterheads are responsible for sending requests on behalf of the whole cluster, i.e. other nodes in the cluster will stay quiet in all the three cases. So, even if CHs have to make more transmissions in the “farthest” case compared to the lesser number of transmissions in the “nearest” case, the overall impact of the additional transmission in the “farthest” case is minimal. This results in almost similar communication overhead curves in the three cases. In other words, even though in the farthest case, the CH may send additional requests, the number of additional requests is very small, and therefore, does not have a significant impact on the communication overhead curves.

#### 5.5.2. Energy Consumption

The results are normalized to be between (0 and 1) using Equation (16).

(16) $$N\left(x_{i}\right)=x_{i}-\frac{min\left(S1,S2,S3\right)}{max\left(S1,S2,S3\right)-min\left(S1,S2,S3\right)}$$

where, S1, S2, and S3 represent the energy consumption matrices of the three cases being compared. *x_i_* is the value to be normalized (*x_i_* ∈ {S1 or S2 or S3}), and min and max are the minimum and maximum value of S1, S2, and S3 combined. 

The farther the CH from the GPS node, the greater the transmission power that may be required. For example, if the farthest node is selected as CH, it may not be able to reach the GPS node with the current transmission range, and therefore will resend a localization request in the next iteration with a doubled transmission range. However, if a node that is nearest to the GPS node is selected as CH, it may reach the GPS node with its smaller transmission power, i.e. in an earlier iteration compared to the farthest case. This justifies the highest energy consumption in the case of the farthest, and the lowest in the case of the nearest.

#### 5.5.3. Localization Coverage

Figure 13 shows the localization coverage for the number of iterations set to 2. As expected, in the case where every cluster selects the node nearest to the GPS as CH, the localization coverage is the highest, whereas it is lowest when every cluster selects the node farthest from the GPS node as CH. The reason is very obvious. When the nearest node is selected, there are more chances that it will reach the GPS node during the current iteration, compared to the lesser chances of reaching the GPS node in the farthest case. Therefore, more nodes are localized in the second iteration in the nearest case, compared to the average case and farthest case.

## 6. Conclusions

The proposed two-stage technique is used to efficiently localize nodes in partitioned networks with the objective of minimizing communication overheads, energy consumption, and localization errors. Stage one estimates the relative positions of all one-hop and multi-hop neighbors of the GPS node. In stage two, which occurs iteratively, partitioned nodes cluster up into one or more clusters based on their communication range. Every cluster dynamically elects a clusterhead that sends localization request on behalf of the whole cluster. Our clustering process is efficient, as it does not require any additional communication, and uses localization requests (sent by unlocalized nodes to request beacons from the GPS node) for clusterhead selection. Cluster merging ensures that the number of transmissions quickly decreases with an increase in the number of iterations thus reducing the contention and communication overheads significantly. This also results in improved energy efficiency of nodes. Besides the clustering technique, our retransmission control technique also plays an important role in reducing communication overheads by restricting unnecessary transmissions. Moreover, by allowing only the GPS-enabled node to send localization beacons we are able to reduce localization errors. This technique reduces error accumulation, compared to the case in which any localized node can send beacons using its estimated coordinates. The simulation results show that our proposed method significantly reduces communication overheads and energy consumption. Localization error is also reduced. However, a slight reduction in localization coverage is observed in the initial iterations. 

As part of the future work, we wish to study the impact of using multiple GPS nodes in our protocol. We also plan to research efficient ways for the GPS node to update its coordinates while staying in contact with the rest of the network. 

## Figures and Tables

**Table 1:** Simulation Parameters.

Parameters	Values
Deployment area	1000 m × 1000 m
Default range	150 m
Number of nodes	20, 40, 60, 80, 100
Number of partitions	1, 2, 3, 4
Packet size	512 bits
Data rate	5000 bps
Error in speed of sound	0.2 m/s [20]
Depth error	0.1 m [20]
Error due to node mobility	0.1 [20]
Anchor node positioning error	±1 m
AUV positioning error	±1 m [14]
Total length of AUV spiral path	5000 m
AUV beacon transmission	50 m
Simulation Runs	100

**Table 2:** Transmission Power VS Range (S2CR 48/47 Acoustic modem) [21]

Energy Consumption (W)	Range (m)
5.5	250
8	500
18	1000
60	Above 1000
